# Algorithms Identifying Patients With Acute Exacerbation of Interstitial Pneumonia and Acute Interstitial Lung Diseases Developed Using Japanese Administrative Data

**DOI:** 10.7759/cureus.53073

**Published:** 2024-01-27

**Authors:** Keisuke Anan, Yuki Kataoka, Kazuya Ichikado, Kodai Kawamura, Yuko Yasuda, Junpei Hisanaga, Tatsuya Nitawaki, Yosuke Yamamoto

**Affiliations:** 1 Department of Healthcare Epidemiology, Kyoto University Graduate School of Medicine / School of Public Health, Kyoto, JPN; 2 Division of Respiratory Medicine, Saiseikai Kumamoto Hospital, Kumamoto, JPN; 3 Clinical Research Support Section, Saiseikai Kumamoto Hospital, Kumamoto, JPN; 4 Department of Systematic Reviewers, Scientific Research Works Peer Support Group, Osaka, JPN; 5 Section of Clinical Epidemiology, Department of Community Medicine, Kyoto University Graduate School of Medicine, Kyoto, JPN; 6 Department of Internal Medicine, Kyoto Min-iren Asukai Hospital, Kyoto, JPN

**Keywords:** administrative data, validation, pulmonary alveolar hemorrhage, acute exacerbation, interstitial lung disease

## Abstract

Background: We aimed to develop algorithms to identify patients with acute exacerbation of interstitial pneumonia and acute interstitial lung diseases using Japanese administrative data.

Methods: This single-center validation study examined diagnostic algorithm accuracies. We included patients >18 years old with at least one claim that was a candidate for acute exacerbation of interstitial pneumonia, acute interstitial lung diseases, and pulmonary alveolar hemorrhage who were admitted to our hospital between January 2016 and December 2021. Diagnoses of these conditions were confirmed by at least two respiratory physicians through a chart review. The positive predictive value was calculated for the created algorithms.

Results: Of the 1,109 hospitalizations analyzed, 285 and 243 were for acute exacerbation of interstitial pneumonia and acute interstitial lung diseases, respectively. As there were only five cases of pulmonary alveolar hemorrhage, we decided not to develop an algorithm for it. For acute exacerbation of interstitial pneumonia, acute interstitial lung diseases, and acute exacerbation of interstitial pneumonia or acute interstitial lung diseases, algorithms with high positive predictive value (0.82, 95% confidence interval: 0.76-0.86; 0.82, 0.74-0.88; and 0.89, 0.85-0.92, respectively) and algorithms with slightly inferior positive predictive value but more true positives (0.81, 0.75-0.85; 0.77, 0.71-0.83; and 0.85, 0.82-0.88, respectively) were developed.

Conclusion: We developed algorithms with high positive predictive value for identifying patients with acute exacerbation of interstitial pneumonia and acute interstitial lung diseases, useful for future database studies on such patients using Japanese administrative data.

## Introduction

Interstitial lung disease (ILD) is the term used to describe a rare and heterogeneous group of diseases with high mortality. Some ILDs have an acute course, while others have a chronic fibrosing course with occasional acute exacerbation (AE), and severe respiratory failure occurs in both types [1]. The poor prognosis associated with AE of idiopathic pulmonary fibrosis (IPF; AE-IPF) is well reported. However, AEs of other fibrotic ILDs are also associated with poor prognoses, although little is known about them [2]. Pulmonary alveolar hemorrhage (PAH) is another disease that requires differentiation from the aforementioned diseases [1]. Each of these diseases requires hospitalization and sometimes results in fatal outcomes. Although two randomized controlled trials (RCTs) exist for one of these conditions, i.e., AE-IPF [3,4], it is not easy to conduct RCTs for such rare diseases and their external validity is limited. Therefore, it is necessary to conduct large observational studies on diverse patient populations utilizing real-world data to complement RCTs [5].

However, large-scale database studies on AE of interstitial pneumonia (IP), acute ILDs, and PAH remain scarce [6]. This scarcity may be attributed to challenges in the accurate diagnosis of these diseases, especially because important imaging information cannot be accessed in database studies. For accurately identifying cases in large-scale database research, it is crucial to conduct validation studies that evaluate the accuracy and reliability of database definitions such as disease names, outcomes, and their algorithms by comparing them with chart reviews [7]. Except for our study on AE-IPF [8] and a study evaluating respiratory disease as a whole [9], there are no validation studies in these fields in Japan. Therefore, the accuracy of the disease names and their algorithms for AE-IP, acute ILDs, and PAH, except for AE-IPF, remain unknown.

We aimed to develop algorithms for identifying patients with AE-IP, acute ILDs, and PAH from Japanese administrative data, using chart review as the reference standard.

This article was previously presented as a poster presentation at the 6th Annual Meeting of the Society for Clinical Epidemiology on November 11, 2023.

## Materials and methods

This study was conducted according to the reporting guidelines for assessing the quality of validation studies using healthcare administrative data (Appendix A) [7].

Setting and patients

We conducted a retrospective, single-center validation study to examine the accuracy of diagnostic algorithms for AE-IP, acute ILDs, and PAH. The study was conducted at the Saiseikai Kumamoto Hospital, which is a 400-bed tertiary teaching hospital located in Kumamoto, Japan. We included patients aged >18 years with at least one claim for the following diagnostic International Classification of Diseases, 10th Revision (ICD-10) codes: J679, J702, J704, J80, J82, J841, J849, J984, M331, M332, or R048 (detailed explanation in the subsequent paragraph) who were admitted to our hospital between January 2016 and December 2021 (Table 1). We did not exclude patients with multiple hospitalizations and considered each hospitalization separately.

**Table 1:** Disease names adopted as inclusion criteria AE, acute exacerbation; AIP, acute interstitial pneumonia; ALI, acute lung injury; ARDS, acute respiratory distress syndrome; ARF, acute respiratory failure; CPFE, combined pulmonary fibrosis and emphysema; DM, dermatomyositis; DAD, diffuse alveolar damage; DPC, diagnosis procedure combination; EP, eosinophilic pneumonia; HP, hypersensitivity pneumonitis; ICD-10, the International Statistical Classification of Diseases and Related Health Problems, Tenth Revision, IIP, idiopathic interstitial pneumonia; ILD, interstitial lung disease; IP, interstitial pneumonia; IPF, idiopathic pulmonary fibrosis; NSIP; nonspecific interstitial pneumonia; OP, organizing pneumonia; PM, polymyositis; UIP, usual interstitial pneumonia *Disease name in main diagnosis, diagnosis that triggered hospitalization, diagnosis using the most medical resources, or diagnosis using the second most medical resources in the DPC data

ICD-10 code*	disease name
J841	IPF
J841	IIP
J841	UIP
J841	pulmonary fibrosis
J841	diffuse IP
J849	IP
J849	NSIP
J841	AIP
J841	DAD
J841	CPFE
J80	ARDS
J82	EP
J984	ALI
J702	acute drug-induced ILDs
J704	drug-induced IP
J679	HP
M331	DM-ILD
M332	PM-ILD
R048	alveolar haemorrhage

We extracted data from the Diagnosis Procedure Combination (DPC), a payment system used in acute care hospitals throughout Japan to reimburse healthcare providers for their medical services [10,11]. Under DPC, providers are paid a fixed amount for each diagnosis-procedure combination, rather than payment according to a fee-for-service model. It is intended to encourage efficient and cost-effective healthcare delivery by reducing unnecessary testing and procedures. Each diagnosis and procedure is assigned a specific DPC code, which is used to determine the amount of money reimbursed to the healthcare provider. The DPC codes are used to classify diagnoses and procedures into categories based on their complexity and resource use. In the DPC, the diagnoses are divided into the following six categories: (1) the main disease, (2) the disease that triggered hospitalization, (3) the disease that required the use of most medical resources, (4) the disease that required the use of second-most medical resources, (5) comorbidities present at admission, and 6) conditions that developed after admission [12]. Disease names belonging to all these categories are assigned ICD-10 codes. Moreover, modifiers such as disease course (acute/chronic), suspicion, and AE can be appended to the disease names. In this study, we used the ICD-10 code for any of the diseases in the first three categories as the algorithm for patient inclusion. We ignored all the claims with "suspicion" attached to them.

**Figure 1:**
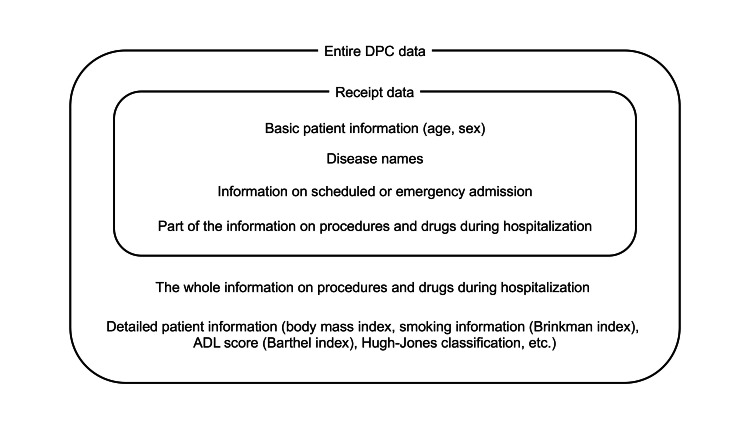
The contents of the entire DPC data and receipt data ADL, activities of daily living; DPC, Diagnosis Procedure Combination

Meanwhile, receipt data, which are part of the DPC content, are submitted to the insurer for billing purposes. These include basic patient information (e.g., age, sex), disease names, certain medical procedures, and drugs (Figure 1). Although these disease names are not as finely classified as the DPC diagnoses, the main disease, disease that triggered hospitalization, and disease using the most medical resources are each flagged for the hospitalized patients. Some information on the administered procedures and drugs is reflected in the receipts, but not necessarily for those that are not calculated according to the DPC system as mentioned above.

We extracted the following data: demographic data (age, sex), body mass index, Brinkman index, Barthel index score, Hugh-Jones classification, disease names, procedures (blood test: D007-28 for Krebs von den Lungen-6 (KL-6) testing and D007-35 for surfactant protein-D (SP-D) testing, imaging: E002-1 for chest CT, oxygen administration information: J024 for oxygen inhalation, J026-4 for high-flow therapy, and J045 for mechanical ventilation), medications (steroid therapy), and additional fee for emergency medical management. 

Reference standard

AE-IP was diagnosed based on the 2016 AE-IPF criteria [13] as follows: (1) previous or concurrent diagnosis of ILD; (2) acute worsening or development of dyspnea, typically within 30 days; (3) high-resolution CT with new bilateral ground-glass opacity and/or consolidation on the background of fibrosis; and (4) deterioration not fully explained by cardiac failure or fluid overload. Acute ILDs included acute IP (AIP), cryptogenic organizing pneumonia (OP), acute hypersensitivity pneumonitis (HP), connective tissue disease-associated ILD (especially idiopathic inflammatory myopathy-associated ILD), acute eosinophilic pneumonia (EP), and drug-associated lung injury [1]. Among these, AIP and cryptogenic OP were diagnosed based on the statement of the American Thoracic Society/European Respiratory Society [14], and acute HP was diagnosed based on the international guidelines of the American Thoracic Society/Japanese Respiratory Society/Latin American Thoracic Association [15]. The diagnosis of idiopathic inflammatory myopathy was based on the European League Against Rheumatism/American College of Rheumatology classification criteria [16]. The diagnosis of acute eosinophilic pneumonia was based on modified Philit criteria [17], and that of drug-associated lung injury was based on criteria reported in a previous study [18]. Meanwhile, because the exact diagnosis of these diseases is sometimes difficult, some cases of acute ILDs were diagnosed through clinical judgment based on the above criteria. If a patient met the criteria for both AE-IP and acute ILDs, AE-IP was considered the diagnosis. For example, patients with drug-associated lung injury were classified as having AE-IP if they had a background of fibrosis on CT and as having acute ILDs if they did not. In the case of PAH, there is no gold standard for diagnosis. Hemoptysis and anemia, along with the findings of imaging (new bilateral infiltrates on chest radiographs/CT) and bronchoalveolar lavage (BAL) studies (progressively hemorrhagic BAL or hemosiderin-laden macrophages) help diagnose PAH [19-21]. However, it was reported that hemoptysis was initially absent in approximately one-third of the cases [20]. Furthermore, BAL is not always possible if a patient has severe respiratory failure. Therefore, PAH was diagnosed clinically based on the aforementioned findings.

Two respiratory specialists preliminarily established the above-mentioned criteria. Diagnoses of AE-IP, acute ILDs, and PAH were confirmed by at least two respiratory physicians based on the aforementioned criteria via chart review. Any conflicts were resolved through discussion. The physicians were blinded to how the claims were coded. 

Developing algorithms

The algorithms were derived using data on the disease names, emergency medical care claims, prescription drugs (corticosteroids), blood tests (KL-6, SP-D), imaging tests (chest CT), and oxygen administration (O2 supplementation, high-flow therapy, and mechanical ventilation). First, the accuracy of each disease name in the inclusion criteria, as well as the combination of these disease names with other information (e.g., the AE modifier and emergency admission flag), were evaluated for the diagnosis of AE-IP, acute ILDs, and PAH. Based on previous studies [8,22], we also evaluated the accuracy of combining each disease name with acute respiratory failure (ARF) (J960). If fewer than 10 patients had any of the three conditions, we discontinued the creation of an algorithm for that disease. Furthermore, for disease combinations with < 10 cases, no additional information on drugs, tests, or procedures was considered. Next, algorithms were created to identify each disease by combining the disease names with a high positive predictive value (PPV). We then developed two sets of algorithms: 1) specific algorithms (narrow algorithms) and 2) algorithms with a marginally lower PPV but higher true positives (TPs) (broad algorithms). When creating the narrow algorithms, we combined disease names with PPVs of 0.8 or above, with higher PPVs receiving preference. Meanwhile, the broad algorithms were developed by merging disease names with PPVs of 0.7 or greater, with a focus on having more TPs. A sample size of approximately 100 is sufficient for validation studies that only attempt to obtain the PPV [23]. Therefore, we created each algorithm using more than 100 cases. 

Statistical analysis

For diagnosis, we evaluated the interobserver agreement between the two respiratory physicians using Gwet’s AC1-index, which is one of the measures of the observer agreement and is less affected by prevalence than kappa statistics [24]. 

For each disease name and its combinations, the PPV was computed as the proportion of TPs to the sum of TPs and false positives. Moreover, the 95% confidence intervals (CIs) for the binomial distribution were computed using an exact method. As a chart review was not performed for patients without the target ICD-10 codes, the sensitivity, specificity, and negative predictive value could not be calculated. Statistical analyses were performed using Stata software (Stata Statistical Software, Release 1, StataCorp LLC, College Station, TX).

Ethics

This study was approved by the Ethics Committee of Saiseikai Kumamoto Hospital (approval number: 1065) and Kyoto University Graduate School and Faculty of Medicine (approval number: R3596). The requirement for written informed consent was waived because of the retrospective nature of the study. We explained this study on the website of our hospital and the patients were given the opportunity to opt out.

## Results

A total of 1,109 hospitalizations were analyzed, including 285 for AE-IP and 243 for acute ILDs. We judged 576 admissions were not for AE-IP, acute ILDs or PAH, of which 94 were not for IP and 482 were for IP but not for an AE or acute ILDs. Of the 842 included patients, 667, 114, 42, 12, 3, 3, and 1 had been admitted once, twice, three times, four times, five times, six times, and seven times, respectively. Only 5 patients had PAH; therefore, we did not develop an algorithm for PAH detection. The patient characteristics are shown in Table 2. The interobserver agreement assessed using Gwet’s AC1-index was 0.78 (95% CI, 0.76-0.81). 

**Table 2:** Patient characteristics AE, acute exacerbation; BMI, body mass index; IP, interstitial pneumonia Data are shown as N (%) or median (interquartile range)

	Total (n = 1109)	AE-IP (n = 285)	Acute ILDs (n = 243)	AE-IP or Acute ILDs (n = 528)
age (years)	72 (67–80)	76 (69–83)	74 (67–83)	75 (68–83)
sex (male)	762 (69%)	216 (76%)	160 (66%)	376 (71%)
BMI (kg/m^2^)	22.5 (19.7–25.2)	22.2 (19.8–24.6)	22.1 (19.2–25.1)	22.1 (19.5–24.9)
missing data	26 (2%)	8 (3%)	8 (3%)	16 (3%)
Brinkman index	400 (0–1000)	440 (0–1000)	375 (0–1170)	400 (0–1006)
Barthel Index	85 (30–100)	40 (15–80)	60 (20–100)	50 (15–95)
Hugh–Jones classification				
0	214 (20%)	63 (22%)	51 (21%)	114 (22%)
1	144 (13%)	6 (2%)	22 (9%)	28 (5%)
2	154 (14%)	11 (4%)	28 (12%)	39 (7%)
3	146 (13%)	26 (9%)	22 (9%)	48 (9%)
4	251 (23%)	92 (32%)	65 (27%)	157 (30%)
5	177 (16%)	80 (28%)	51 (21%)	131 (25%)
missing data	23 (2%)	7 (2%)	4 (2%)	11 (2%)
In-hospital death	154 (14%)	74 (26%)	28 (12%)	102 (19%)

The PPVs with 95% CIs for each disease name are shown in Table 3. Several disease names (such as diffuse IP, nonspecific IP, diffuse alveolar damage, and acute lung injury) occurred in zero patients. In addition, Table 4 shows the disease names with additional information on steroid therapy, and Table 5 shows those with information on KL-6 and SP-D tests, chest CT, O2 supplementation, high-flow therapy, and mechanical ventilation. We included only additional information on steroid therapy in the algorithm because including the other information reduced the number of patients and the information did not outweigh the information on steroid therapy. In the receipt data, the recorded percentages of blood tests, imaging tests, procedures, and drugs were low, even though they were actually performed in clinical practice (KL-6: 37/771, 5%; SP-D: 56/574, 10%; chest CT: 34/437, 8%; oxygen inhalation: 48/776, 6%; high-flow therapy: 5/87, 6%; mechanical ventilation: 21/174, 12%; and steroid therapy: 101/668, 15%). However, this information was reflected in the DPC dataset. Therefore, we decided to create two groups of algorithms, depending on whether additional information on steroid therapy was available: 1) when only receipt data were available and 2) when data from the entire DPC dataset were available. In cases of prolonged hospitalization, where multiple hospitalization receipts could have been generated, the receipts from the last month were used. The PPVs and 95% CIs for each disease, generated using receipt data alone, are shown in Table 6.

**Table 3:** The positive predictive value (PPV) and 95% confidence intervals (CI) of patients diagnosed with acute exacerbation of interstitial pneumonia or acute interstitial lung diseases within each algorithm AE, acute exacerbation; AIP, acute interstitial pneumonia; ALI, acute lung injury; ARDS, acute respiratory distress syndrome; ARF, acute respiratory failure; CPFE, combined pulmonary fibrosis and emphysema; DAD, diffuse alveolar damage; DM, Dermatomyositis; EP, eosinophilic pneumonia; HP, hypersensitivity pneumonitis; IIP, idiopathic interstitial pneumonia; ILD, interstitial lung disease; IP, interstitial pneumonia; IPF, idiopathic pulmonary fibrosis; NSIP; nonspecific interstitial pneumonia; OP, organizing pneumonia; PM, Polymyositis; PPV, positive predictive value; TP; true positive; UIP, usual interstitial pneumonia

Disease names and their combinations		AE-IP		Acute ILDs		AE-IP or Acute ILDs	
	Total (n)	TP (n)	PPV (95% CI)	TP (n)	PPV (95% CI)	TP (n)	PPV (95% CI)
IPF + AE	34	24	0.71 (0.53-0.85)	0	0 (0-0.10)	24	0.71 (0.53-0.85)
IPF + emergency admission	38	4	0.11 (0.029-0.25)	0	0 (0-0.093)	4	0.11 (0.029-0.25)
IPF + ARF	8	1	0.13 (0.0032-0.53)	0	0 (0-0.37)	1	0.13 (0.0032-0.53)
IIP + AE	61	53	0.87 (0.76-0.94)	1	0.016 (0.00042-0.088)	54	0.89 (0.78-0.95)
IIP + emergency admission	15	0	0 (0-0.22)	3	0.20 (0.043-0.48)	3	0.20 (0.043-0.48)
IIP + ARF	3	0	0 (0-0.71)	3	1 (0.29-1)	3	1 (0.29-1)
UIP + AE	1	0	0 (0-0.98)	0	0 (0-0.98)	0	0 (0-0.98)
UIP + emergency admission	1	0	0 (0-0.98)	0	0 (0-0.98)	0	0 (0-0.98)
UIP + ARF	1	0	0 (0-0.98)	0	0 (0-0.98)	0	0 (0-0.98)
pulmonary fibrosis + AE	13	8	0.62 (0.32-0.86)	2	0.15 (0.019-0.45)	10	0.77 (0.46-0.95)
pulmonary fibrosis + emergency admission	8	0	0 (0-0.37)	3	0.38 (0.085-0.76)	3	0.38 (0.085-0.76)
pulmonary fibrosis + ARF	6	0	0 (0-0.46)	3	0.50 (0.12-0.88)	3	0.50 (0.12-0.88)
IP + AE	169	138	0.82 (0.75-0.87)	9	0.053 (0.025-0.099)	147	0.87 (0.81-0.92)
IP + emergency admission	82	12	0.15 (0.078-0.24)	33	0.40 (0.30-0.52)	45	0.55 (0.43-0.66)
IP + ARF	43	4	0.093 (0.026-0.22)	27	0.63 (0.47-0.77)	31	0.72 (0.56-0.85)
CPFE + AE	3	2	0.67 (0.094-0.99)	0	0 (0-0.71)	2	0.67 (0.094-0.99)
CPFE + emergency admission	1	0	0 (0-0.98)	0	0 (0-0.98)	0	0 (0-0.98)
CPFE + ARF	0	0	0	0	0	0	0
OP	44	1	0.023 (0.00058-0.12)	32	0.73 (0.57-0.85)	33	0.75 (0.60-0.87)
OP + AE	2	0	0 (0-0.84)	2	1 (0.16-1)	2	1 (0.16-1)
OP + emergency admission	33	1	0.030 (0.00077-0.16)	26	0.79 (0.61-0.91)	27	0.82 (0.65-0.93)
OP + ARF	16	0	0 (0-0.21)	10	0.63 (0.35-0.85)	10	0.63 (0.35-0.85)
HP	18	1	0.056 (0.0014-0.27)	4	0.22 (0.064-0.48)	5	0.28 (0.097-0.53)
HP + AE	2	2	1 (0.16-1)	0	0 (0-0.84)	2	1 (0.16-1)
HP + emergency admission	5	1	0.20 (0.0051-0.72)	2	0.40 (0.053-0.85)	3	0.60 (0.15-0.95)
HP + ARF	4	1	0.25 (0.0063-0.81)	2	0.50 (0.068-0.93)	3	0.75 (0.19-0.99)
NSIP	5	0	0 (0-0.52)	0	0 (0-0.52)	0	0 (0-0.52)
AIP	9	2	0.22 (0.028-0.60)	7	0.78 (0.40-0.97)	9	1 (0.66-1)
ARDS	102	15	0.14 (0.085-0.23)	29	0.28 (0.20-0.38)	44	0.43 (0.33-0.53)
ARDS + emergency admission	96	13	0.14 (0.074-0.22)	28	0.29 (0.20-0.39)	41	0.43 (0.33-0.53)
ARDS + ARF	62	10	0.16 (0.080-0.28)	21	0.34 (0.22-0.47)	31	0.5 (0.37-0.63)
acute drug-induced ILDs	74	10	0.14 (0.067-0.23)	61	0.82 (0.72-0.90)	71	0.96 (0.89-0.99)
acute drug-induced ILDs + emergency admission	63	8	0.13 (0.056-0.23)	52	0.83 (0.71-0.91)	60	0.95 (0.87-0.99)
acute drug-induced ILDs + ARF	39	7	0.18 (0.075-0.34)	32	0.82 (0.66-0.92)	39	1 (0.91-1)
drug-induced IP	65	11	0.17 (0.088-0.28)	47	0.72 (0.60-0.83)	58	0.89 (0.79-0.96)
drug-induced IP + emergency admission	54	7	0.13 (0.054-0.25)	43	0.80 (0.66-0.89)	50	0.93 (0.82-0.98)
drug-induced IP + ARF	33	6	0.18 (0.070-0.35)	26	0.79 (0.61-0.91)	32	0.97 (0.84-0.999)
EP	7	1	0.14 (0.0036-0.58)	6	0.86 (0.42-0.996)	7	1 (0.59-1)
PM-ILD	1	0	0 (0-0.98)	0	0 (0-0.98)	0	0 (0-0.98)
DM-ILD	2	1	0.5 (0.013-0.99)	1	0.5 (0.013-0.99)	2	1 (0.16-1)
alveolar haemorrhage	8	0	0 (0-0.37)	0	0 (0-0.37)	0	0 (0-0.37)

**Table 4:** The positive predictive value (PPV) and 95% confidence intervals (CI) of patients diagnosed with acute exacerbation of interstitial pneumonia or acute interstitial lung diseases within each algorithm AE, acute exacerbation; AIP, acute interstitial pneumonia; ALI, acute lung injury; ARDS, acute respiratory distress syndrome; ARF, acute respiratory failure; CPFE, combined pulmonary fibrosis and emphysema; DAD, diffuse alveolar damage; DM, Dermatomyositis; EP, eosinophilic pneumonia; HP, hypersensitivity pneumonitis; IIP, idiopathic interstitial pneumonia; ILD, interstitial lung disease; IP, interstitial pneumonia; IPF, idiopathic pulmonary fibrosis; NSIP; nonspecific interstitial pneumonia; OP, organizing pneumonia; PM, Polymyositis; PPV, positive predictive value; TP; true positive; UIP, usual interstitial pneumonia *steroid pulse therapy, ≥ 500 mg/day of methylprednisolone equivalent; high dose, ≥ 1.0 mg/kg/day or ≥ 50 mg/day (if body weight data were not available) of prednisolone equivalent; moderate dose, 0.5−1.0 mg/kg/day or 25−50 mg/day (if body weight data were not available) of prednisolone equivalent

		AE-IP		Acute ILDs		AE-IP or Acute ILDs	
disease names	Total (n)	TP (n)	PPV (95% CI)	TP (n)	PPV (95% CI)	TP (n)	PPV (95% CI)
IPF + AE + steroid pulse*	15	13	0.87 (0.60-0.98)	0	0 (0-0.22)	13	0.87 (0.60-0.98)
IPF + AE + pulse/high dose steroid*	15	13	0.87 (0.60-0.98)	0	0 (0-0.22)	13	0.87 (0.60-0.98)
IPF + AE + pulse/high/moderate dose steroid*	23	20	0.87 (0.66-0.97)	0	0 (0-0.15)	20	0.87 (0.66-0.97)
IPF + emergency admission + steroid pulse*	1	1	1 (0.025-1)	0	0 (0-0.98)	1	1 (0.025-1)
IPF + emergency admission + pulse/high dose steroid*	1	1	1 (0.025-1)	0	0 (0-0.98)	1	1 (0.025-1)
IPF + emergency admission + pulse/high/moderate dose steroid*	3	3	1 (0.29-1)	0	0 (0-0.71)	3	1 (0.29-1)
IPF + ARF + pulse/high/moderate dose steroid*	0	0	0	0	0	0	0
IIP + AE + steroid pulse*	38	35	0.92 (0.79-0.98)	1	0.026 (0.00067-0.14)	36	0.94 (0.82-0.99)
IIP + AE + pulse/high dose steroid*	41	38	0.93 (0.80-0.98)	1	0.024 (0.00061-0.13)	39	0.95 (0.83-0.99)
IIP + AE + pulse/high/moderate dose steroid*	50	46	0.92 (0.81-0.98)	1	0.02 (0.00051-0.11)	47	0.94 (0.83-0.99)
IIP + emergency admission + pulse/high/moderate dose steroid*	1	0	0 (0-0.98)	1	1 (0.025-1)	1	1 (0.025-1)
IIP + ARF + pulse/high/moderate dose steroid*	1	0	0 (0-0.98)	1	1 (0.025-1)	1	1 (0.025-1)
pulmonary fibrosis + AE + steroid pulse*	3	2	0.67 (0.094-0.99)	1	0.33 (0.0084-0.91)	3	1 (0.29-1)
pulmonary fibrosis + AE + pulse/high dose steroid*	3	2	0.67 (0.094-0.99)	1	0.33 (0.0084-0.91)	3	1 (0.29-1)
pulmonary fibrosis + AE + pulse/high/moderate dose steroid*	6	4	0.67 (0.22-0.96)	1	0.17(0.0042-0.64)	5	0.83 (0.36-0.996)
pulmonary fibrosis + emergency admission + steroid pulse*	1	0	0 (0-0.98)	1	1 (0.025-1)	1	1 (0.025-1)
pulmonary fibrosis + emergency admission + pulse/high dose steroid*	2	0	0 (0-0.84)	2	1 (0.16-1)	2	1 (0.16-1)
pulmonary fibrosis + emergency admission + pulse/high/moderate dose steroid*	4	0	0 (0-0.60)	2	0.50 (0.068-0.93)	2	0.50 (0.068-0.93)
pulmonary fibrosis + ARF + steroid pulse*	1	0	0 (0-0.98)	1	1 (0.025-1)	1	1 (0.025-1)
pulmonary fibrosis + ARF + pulse/high dose steroid*	2	0	0 (0-0.84)	2	1 (0.16-1)	2	1 (0.16-1)
pulmonary fibrosis + ARF+ pulse/high/moderate dose steroid*	3	0	0 (0-0.71)	2	0.67 (0.094-0.99)	2	0.67 (0.094-0.99)
IP + AE + steroid pulse*	96	90	0.94 (0.87-0.98)	4	0.042 (0.011-0.10)	94	0.98 (0.93-0.997)
IP + AE + pulse/high dose steroid*	109	99	0.91 (0.84-0.96)	5	0.046 (0.015-0.10)	104	0.95 (0.90-0.98)
IP + AE + pulse/high/moderate dose steroid*	128	113	0.88 (0.81-0.93)	8	0.063 (0.027-0.12)	121	0.95 (0.89-0.98)
IP + emergency admission + steroid pulse*	12	1	0.083 (0.0021-0.38)	9	0.75 (0.43-0.95)	10	0.83 (0.51-0.98)
IP + emergency admission + pulse/high dose steroid*	26	4	0.15 (0.043-0.35)	18	0.69 (0.48-0.86)	22	0.85 (0.65-0.96)
IP + emergency admission + pulse/high/moderate dose steroid*	36	5	0.14 (0.047-0.29)	24	0.67 (0.49-0.81)	29	0.81 (0.64-0.92)
IP + ARF + steroid pulse	11	0	0 (0-0.28)	9	0.82 (0.48-0.98)	9	0.82 (0.48-0.98)
IP + ARF + pulse/high dose steroid*	22	3	0.14 (0.029-0.35)	16	0.73 (0.50-0.89)	19	0.86 (0.65-0.97)
IP + ARF + pulse/high/moderate dose steroid*	30	4	0.13 (0.038-0.31)	22	0.73 (0.54-0.88)	26	0.87 (0.69-0.96)
OP + steroid pulse*	1	0	0 (0-0.98)	1	1 (0.025-1)	1	1 (0.025-1)
OP + pulse/high dose steroid*	3	0	0 (0-0.71)	3	1 (0.29-1)	3	1 (0.29-1)
OP + pulse/high/moderate dose steroid*	9	0	0 (0-0.34)	8	0.89 (0.52-0.997)	8	0.89 (0.52-0.997)
acute drug-induced ILDs + steroid pulse*	23	5	0.22 (0.075-0.44)	18	0.78 (0.56-0.93)	23	1 (0.85-1)
acute drug-induced ILDs + pulse/high dose steroid*	29	6	0.21 (0.080-0.40)	23	0.79 (0.60-0.92)	29	1 (0.88-1)
acute drug-induced ILDs + pulse/high/moderate dose steroid*	41	8	0.20 (0.088-0.35)	33	0.80 (0.65-0.91)	41	1 (0.91-1)
acute drug-induced ILDs + ARF + steroid pulse*	17	3	0.18 (0.038-0.43)	14	0.82 (0.57-0.96)	17	1 (0.80-1)
acute drug-induced ILDs + ARF + pulse/high dose steroid*	20	4	0.2 (0.057-0.44)	16	0.8 (0.56-0.94)	20	1 (0.83-1)
acute drug-induced ILDs + ARF + pulse/high/moderate dose steroid*	26	5	0.19 (0.066-0.39)	21	0.81 (0.61-0.93)	26	1 (0.87-1)
drug-induced IP + steroid pulse*	21	6	0.29 (0.11-0.52)	15	0.71 (0.48-0.89)	21	1 (0.84-1)
drug-induced IP + pulse/high dose steroid*	25	7	0.28 (0.12-0.49)	18	0.72 (0.51-0.88)	25	1 (0.86-1)
drug-induced IP + pulse/high/moderate dose steroid*	33	8	0.24 (0.11-0.42)	24	0.73 (0.54-0.87)	32	0.97 (0.84-0.999)
drug-induced IP + ARF + steroid pulse*	18	5	0.28 (0.097-0.53)	13	0.72 (0.47-0.90)	18	1 (0.81-1)
drug-induced IP + ARF + pulse/high dose steroid*	19	5	0.26 (0.091-0.51)	14	0.74 (0.49-0.91)	19	1 (0.82-1)
drug-induced IP + ARF + pulse/high/moderate dose steroid*	23	6	0.26 (0.10-0.48)	17	0.74 (0.52-0.90)	23	1 (0.85-1)

**Table 5:** The positive predictive value (PPV) and 95% confidence intervals (CI) of patients diagnosed with acute exacerbation of interstitial pneumonia or acute interstitial pneumonia within each disease name and procedure/test AE, acute exacerbation; ARF, acute respiratory failure; CT, computed tomography; HFNC, high-flow nasal cannula; IIP, idiopathic interstitial pneumonia; ILD, interstitial lung disease; IP, interstitial pneumonia; IPF, idiopathic pulmonary fibrosis; KL-6, Krebs von den Lungen-6; MV, mechanical ventilation; O2, oxygen therapy; OP, organizing pneumonia; PPV, positive predictive value; SP-D, surfactant protein-D; TP, true positive

Disease names and procedures/tests		AE-IP		Acute ILDs		AE-IP or Acute ILDs	
	Total (n)	TP (n)	PPV (95% CI)	TP (n)	PPV (95% CI)	TP (n)	PPV (95% CI)
IPF + AE + O_2_	31	21	0.68 (0.49-0.83)	0	0 (0-0.11)	21	0.68 (0.49-0.83)
IPF + AE + HFNC	6	4	0.67 (0.22-0.96)	0	0 (0-0.46)	4	0.67 (0.22-0.96)
IPF + AE + MV	5	5	1 (0.48-1)	0	0 (0-0.52)	5	1 (0.48-1)
IPF + AE + KL-6	33	23	0.70 (0.51-0.84)	0	0 (0-0.11)	23	0.70 (0.51-0.84)
IPF + AE + SP-D	18	14	0.78 (0.52-0.94)	0	0 (0-0.19)	14	0.78 (0.52-0.94)
IPF + AE + CT	15	12	0.8 (0.52-0.96)	0	0 (0-0.22)	12	0.8 (0.52-0.96)
IPF + ARF + O_2_	23	12	0.52 (0.31-0.73)	0	0 (0-0.15)	12	0.52 (0.31-0.73)
IPF + ARF + HFNC	4	2	0.5 (0.068-0.93)	0	0 (0-0.60)	2	0.5 (0.068-0.93)
IPF + ARF + MV	3	3	1 (0.29-1)	0	0 (0-0.71)	3	1 (0.29-1)
IPF + ARF + KL-6	25	14	0.56 (0.35-0.76)	0	0 (0-0.14)	14	0.56 (0.35-0.76)
IPF + ARF + SP-D	16	10	0.63-0.35-0.85)	0	0 (0-0.21)	10	0.63-0.35-0.85)
IPF + ARF + CT	9	7	0.78 0.40-0.97)	0	0 (0-0.34)	7	0.78 (0.40-0.97)
IIP + AE + O_2_	54	47	0.87 (0.75-0.95)	1	0.019 (0.00047-0.099)	48	0.89 (0.77-0.96)
IIP + AE + HFNC	11	10	0.91 (0.59-0.998)	1	0.091 (0.0022-0.41)	11	1 (0.72-1)
IIP + AE + MV	9	8	0.89 (0.52-0.997)	0	0 (0-0.34)	8	0.89 (0.52-0.997)
IIP + AE + KL-6	55	50	0.91 0.80-0.97)	1	0.018 (0.00046-0.097)	51	0.93 (0.82-0.98)
IIP + AE + SP-D	48	46	0.96 (0.86-0.99)	0	0 (0-0.074)	46	0.96 (0.86-0.99)
IIP + AE + CT	35	33	0.94 (0.81-0.99)	0	0 (0-0.10)	33	0.94 (0.81-0.99)
IIP + ARF + O_2_	28	22	0.79 (0.59-0.92)	4	0.14 (.040-0.33)	26	0.93 (0.76-0.99)
IIP + ARF + HFNC	6	5	0.83 (0.36-0.996)	1	0.17 (0.0042-0.64)	6	1 (0.54-1)
IIP + ARF + MV	7	7	1 (0.59-1)	0	0 (0-0.41)	7	1 (0.59-1)
IIP + ARF + KL-6	28	23	0.82 (0.63-0.94)	4	0.14 (0.040-0.33)	27	0.96 (0.82-0.999)
IIP + ARF + SP-D	26	23	0.88 (0.70 0.98)	3	0.12 (0.024-0.30)	26	1 (0.87-1)
IIP + ARF + CT	15	14	0.93 (0.68-0.998)	1	0.067 (0.0017-0.32)	15	1 (0.78-1)
IP + AE + O_2_	139	116	0.83 (0.76-0.89)	6	0.043 (0.016-0.091)	122	0.88 (0.81-0.93)
IP + AE + HFNC	21	20	0.95 (0.76-0.999)	0	0 (0-0.16)	20	0.95 (0.76-0.999)
IP + AE + MV	41	35	0.85 (0.71-0.94)	1	0.024 (0.00062-0.13)	36	0.88 (0.74-0.96)
IP + AE + KL-6	159	131	0.82 (0.76-0.88)	8	0.050 (0.022-0.097)	139	0.87 (0.81 0.92)
IP + AE + SP-D	134	113	0.84 (0.77-0.90)	9	0.067-(0.031-0.12)	122	0.91(0.85-0.95)
IP + AE + CT	101	91	0.90 (0.83-0.95)	4	0.040 (0.011-0.098)	95	0.94-(0.88-0.98)
IP + ARF + O_2_	135	88	0.65 (0.57-0.73)	29	0.21 (0.15-0.29)	117	0.87 (0.80-0.92)
IP + ARF + HFNC	22	17	0.77 (0.55-0.92)	3	0.14 (0.029-0.35)	20	0.91 (0.71-0.99)
IP + ARF + MV	42	30	0.71 (0.55-0.84)	5	0.12 (0.040-0.26)	35	0.83 (0.69-0.93)
IP + ARF + KL-6	141	94	0.67 (0.58-0.74)	31	0.22 (0.15-0.30)	125	0.89 (0.82-0.93)
IP + ARF + SP-D	124	85	0.69 (0.60-0.77)	28	0.23 (0.16-0.31)	113	0.91 (0.85-0.95)
IP + ARF + CT	89	66	0.74 (0.64-0.83)	18	0.20 (0.12-0.30)	84	0.94 (0.87-0.98)
OP + O_2_	39	0	0 (0-0.090)	30	0.77 (0.61-0.89)	30	0.77 (0.61-0.89)
OP + HFNC	4	0	0 (0-0.60)	2	0.5 (0.068-0.93)	2	0.5 (0.068-0.93)
OP + MV	0	0	0	0	0	0	0
OP + KL-6	32	1	0.031 (0.00079-0.16)	27	0.84 (0.67-0.95)	28	0.88 (0.71-0.96)
OP + SP-D	25	1	0.04 (0.001-0.20)	22	0.88 (0.69-0.97)	23	0.92 (0.74-0.99)
OP + CT	27	1	0.037 (0.00094-0.19)	22	0.81 (0.62-0.94)	23	0.85 (0.66-0.96)
acute drug-induced ILDs + O_2_	58	5	0.086 (0.029-0.19)	51	0.88 (0.77-0.95)	56	0.97 (0.88-0.996)
acute drug-induced ILDs + HFNC	8	2	0.25 (0.032-0.65)	6	0.75 (0.35-0.97)	8	1 (0.63-1)
acute drug-induced ILDs + MV	5	1	0.2 (0.0051-0.72)	4	0.8 (.28-0.99)	5	1 (0.48-1)
acute drug-induced ILDs + KL-6	66	8	0.12 (0.054-0.22)	55	0.83 (0.72-0.91)	63	0.95 (0.87-0.99)
acute drug-induced ILDs + SP-D	43	8	0.19 (0.084-0.33)	33	0.77(0.61-0.88)	41	0.95(0.84-0.99)
acute drug-induced ILDs + CT	44	5	0.11 (0.038-0.25)	37	0.84(0.70-0.93)	42	0.95 (0.85-0.99)
acute drug-induced ILDs + ARF + O_2_	36	5	0.14 (0.047-0.29)	31	0.86 (0.71-0.95)	36	1 (0.90-1)
acute drug-induced ILDs + ARF + HFNC	8	2	0.25 (0.032-0.65)	6	0.75 (0.35-0.97)	8	1 (0.63-1)
acute drug-induced ILDs + ARF + MV	4	1	0.25 (0.0063-0.81)	3	0.75 (0.19-0.99)	4	1 (0.40-1)
acute drug-induced ILDs + ARF + KL-6	37	7	0.19 (0.080-0.35)	30	0.81 (0.65-0.92)	37	1 (0.91-1)
acute drug-induced ILDs + ARF + SP-D	28	7	0.25 (0.11-0.45)	21	0.75 (0.55-0.89)	28	1 (0.88-1)
acute drug-induced ILDs + ARF + CT	24	4	0.17 (0.047-0.37)	20	0.83 (0.63-0.95)	24	1 (0.86-1)
drug-induced IP + O_2_	55	10	0.18 (0.091-0.31)	38	0.69 (0.55-0.81)	48	0.87 (0.76-0.95)
drug-induced IP + HFNC	3	1	0.33 (0.0084-0.91)	2	0.67(0.094-0.99)	3	1 (0.29-1)
drug-induced IP + MV	4	0	0 (0-0.60)	4	1 (0.40-1)	4	1 (0.40-1)
drug-induced IP + KL-6	54	9	0.17 (0.079-0.29)	40	0.74 (0.60-0.85)	49	0.91 (0.80-0.97)
drug-induced IP + SP-D	41	8	0.20 (0.088-0.35)	29	0.71 (0.54-0.84)	37	0.90 (0.77-0.97)
drug-induced IP + CT	38	7	0.18 (0.077-0.34)	27	0.71 (0.54-0.85)	34	0.89 (0.75-0.97)
drug-induced IP + ARF + O_2_	33	6	0.18 (0.070-0.35)	26	0.79 (0.61-0.91)	32	0.97 (0.84-0.999)
drug-induced IP + ARF + HFNC	3	1	0.33 (0.0084-0.91)	2	0.67(0.094-0.99)	3	1 (0.29-1)
drug-induced IP + ARF + MV	3	0	0 (0-0.71)	3	1 (0.29-1)	3	1 (0.29-1)
drug-induced IP + ARF + KL-6	31	6	0.19 (0.075-0.37)	25	0.81 (0.63-0.93)	31	1 (0.89-1)
drug-induced IP + ARF + SP-D	25	6	0.24 (0.094-0.45)	19	0.76 (0.55-0.91)	25	1 (0.86-1)
drug-induced IP + ARF + CT	19	3	0.16 (0.034-0.40)	16	0.84 (0.60-0.97)	19	1 (0.82-1)

**Table 6:** The positive predictive value (PPV) and 95% confidence intervals (CI) of patients diagnosed with acute exacerbation of interstitial pneumonia or acute interstitial lung diseases within each algorithm AE, acute exacerbation; AIP, acute interstitial pneumonia; ALI, acute lung injury; ARDS, acute respiratory distress syndrome; ARF, acute respiratory failure; CPFE, combined pulmonary fibrosis and emphysema; DAD, diffuse alveolar damage; DM, Dermatomyositis; EP, eosinophilic pneumonia; HP, hypersensitivity pneumonitis; IIP, idiopathic interstitial pneumonia; ILD, interstitial lung disease; IP, interstitial pneumonia; IPF, idiopathic pulmonary fibrosis; NSIP; nonspecific interstitial pneumonia; OP, organizing pneumonia; PM, Polymyositis; PPV, positive predictive value; TP; true positive; UIP, usual interstitial pneumonia

Disease names and their combinations		AE-IP		Acute ILDs		AE-IP or Acute ILDs	
	Total (n)	TP (n)	PPV (95% CI)	TP (n)	PPV (95% CI)	TP (n)	PPV (95% CI)
IPF + AE	32	23	0.72 (0.53-0.86)	0	0 (0-0.10)	23	0.72 (0.53-0.86)
IPF + emergency admission	39	5	0.13 (0.043-0.27)	0	0 (0-0.090)	5	0.13 (0.043-0.27)
IPF + ARF	9	2	0.22 (0.028-0.60)	0	0 (0-0.34)	2	0.22 (0.028-0.60)
IIP + AE	63	55	0.86 (0.75-0.93)	1	0.016 (0.00040-0.085)	55	0.87 (0.77-0.94)
IIP + emergency admission	15	0	0 (0-0.22)	3	0.20 (0.043-0.48)	3	0.20 (0.043-0.48)
IIP + ARF	3	0	0 (0-0.71)	3	1 (0.29-1)	3	1 (0.29-1)
UIP + AE	1	0	0 (0-0.98)	0	0 (0-0.98)	0	0 (0-0.98)
UIP + emergency admission	1	0	0 (0-0.98)	0	0 (0-0.98)	0	0 (0-0.98)
UIP + ARF	1	0	0 (0-0.98)	0	0 (0-0.98)	0	0 (0-0.98)
pulmonary fibrosis + AE	12	8	0.67 (0.35-0.90)	1	0.083 (0.0021-0.38)	9	0.75 (0.43-0.95)
pulmonary fibrosis + emergency admission	10	1	0.10 (0.0025-0.45)	4	0.40 (0.12-0.74)	5	0.50 (0.19-0.81)
pulmonary fibrosis + ARF	7	0	0 (0-0.41)	4	0.57 (0.18-0.90)	4	0.57 (0.18-0.90)
IP + AE	171	138	0.81 (0.74-0.86)	11	0.064 (0.033-0.11)	149	0.87 (0.81-0.92)
IP + emergency admission	89	15	0.17 (0.098-0.26)	33	0.37 (0.27-0.48)	48	0.54 (0.43-0.65)
IP + ARF	47	6	0.13 (0.048-0.26)	27	0.57 (0.42-0.72)	33	0.70 (0.55-0.83)
CPFE + AE	2	1	0.5 (0.013-0.99)	0	0 (0-0.84)	1	0.5 (0.013-0.99)
CPFE + emergency admission	1	0	0 (0-0.98)	0	0 (0-0.98)	0	0 (0-0.98)
CPFE + ARF	0	0	0	0	0	0	0
OP	44	1	0.023 (0.00058-0.12)	32	0.73 (0.57-0.85)	33	0.75 (0.60-0.87)
OP + AE	2	0	0 (0-0.84)	2	1 (0.16-1)	2	1 (0.16-1)
OP + emergency admission	33	1	0.030 (0.00077-0.16)	26	0.79 (0.61-0.91)	27	0.82 (0.65-0.93)
OP + ARF	16	0	0 (0-0.21)	10	0.63 (0.35-0.85)	10	0.63 (0.35-0.85)
HP	18	1	0.056 (0.0014-0.27)	4	0.22 (0.064-0.48)	5	0.28 (0.097-0.53)
HP + AE	2	2	1 (0.16-1)	0	0 (0-0.84)	2	1 (0.16-1)
HP + emergency admission	5	1	0.20 (0.0051-0.72)	2	0.40 (0.053-0.85)	3	0.60 (0.15-0.95)
HP + ARF	4	1	0.25 (0.0063-0.81)	2	0.50 (0.068-0.93)	3	0.75 (0.19-0.99)
NSIP	5	0	0 (0-0.52)	0	0 (0-0.52)	0	0 (0-0.52)
AIP	9	2	0.22 (0.028-0.60)	7	0.78 (0.40-0.97)	9	1 (0.66-1)
ARDS	101	15	0.15 (0.086-0.23)	28	0.28 (0.19-0.38)	43	0.43 (0.33-0.53)
ARDS + emergency admission	95	13	0.14 (0.075-0.22)	27	0.28 (0.20-0.39)	40	0.42 (0.32-0.53)
ARDS + ARF	62	10	0.16 (0.080-0.28)	21	0.34 (0.22-0.47)	31	0.5 (0.37-0.63)
acute drug-induced ILDs	72	10	0.14 (0.069-0.24)	59	0.82 (0.71-0.90)	69	0.96 (0.88-0.99)
acute drug-induced ILDs + emergency admission	62	8	0.13 (0.057-0.24)	51	0.82 (0.70-0.91)	59	0.95 (0.87-0.99)
acute drug-induced ILDs + ARF	39	7	0.18 (0.075-0.34)	32	0.82 (0.66-0.92)	39	1 (0.91-1)
drug-induced IP	65	10	0.15 (0.076-0.26)	48	0.74 (0.61-0.84)	58	0.89 (0.79-0.96)
drug-induced IP + emergency admission	54	6	0.11 (0.042-0.23)	44	0.81 (0.69-0.91)	50	0.93 (0.82-0.98)
drug-induced IP + ARF	32	5	0.16 (0.053-0.33)	26	0.81 (0.64-0.93)	31	0.97 (0.84-0.999)
EP	7	1	0.14 (0.0036-0.58)	6	0.86 (0.42-0.996)	7	1 (0.59-1)
PM-ILD	1	0	0 (0-0.98)	0	0 (0-0.98)	0	0 (0-0.98)
DM-ILD	2	1	0.5 (0.013-0.99)	1	0.5 (0.013-0.99)	2	1 (0.16-1)
alveolar haemorrhage	8	0	0 (0-0.37)	0	0 (0-0.37)	0	0 (0-0.37)

Algorithms for AE-IP

Narrow Algorithm Without Corticosteroid Information

Several disease names that had PPVs higher than 0.8 for AE-IP were identified, including those with AE modifiers (AE-idiopathic IP, AE-IP, and AE-HP). Combining these disease names, a narrow algorithm without corticosteroid information with a PPV of 0.82 (95% CI, 0.76-0.86) was derived (Table 7). 

**Table 7:** Final algorithms AE, acute exacerbation; AIP, acute interstitial pneumonia; DM, Dermatomyositis; EP, eosinophilic pneumonia; HP, hypersensitivity pneumonitis; IIP, idiopathic interstitial pneumonia; ILD, interstitial lung disease; IP, interstitial pneumonia; IPF, idiopathic pulmonary fibrosis; OP, organizing pneumonia; PM, Polymyositis; PPV, positive predictive value; TP; true positive *steroid pulse therapy, ≥ 500 mg/day of methylprednisolone equivalent; high dose, ≥ 1.0 mg/kg/day or ≥ 50 mg/day (if body weight data were not available) of prednisolone equivalent; moderate dose, 0.5−1.0 mg/kg/day or 25−50 mg/day (if body weight data were not available) of prednisolone equivalent

Algorithm	Combination of disease name	Total (n)	TP (n)	PPV (95% CI)
Narrow algorithm without corticosteroid information				
AE-IP	(IIP OR IP OR HP) + AE	236	193	0.82 (0.76-0.86)
Acute ILDs	(OP + AE) OR acute drug-induced ILDs OR (drug-induced IP + emergency admission) OR EP	133	109	0.82 (0.74-0.88)
AE-IP or Acute ILDs	(IIP + (AE OR ARF)) OR ((IP OR HP) + AE) OR (OP + (AE OR emergency admission) OR AIP OR acute drug-induced ILDs OR drug-induced IP OR EP OR DM-ILD	423	376	0.89 (0.85-0.92)
Narrow algorithm with corticosteroid information				
AE-IP	((IIP OR IP OR HP) + AE) OR (IPF + (AE OR emergency admission) + pulse/high/moderate dose steroid*)	255	213	0.84 (0.78-0.88)
Acute ILDs	(OP + AE) OR acute drug-induced ILDs OR (drug-induced IP + emergency admission) OR EP OR (IIP + (emengency adimission OR ARF) + pulse/high/moderate dose steroid*) OR (pulmonary fibrosis + (emengency adimission OR ARF) + pulse/high dose steroid*) OR (IP + ARF + steroid pulse) OR (OP + pulse/high/moderate dose steroid*)	158	129	0.82 (0.75-0.87)
AE-IP or Acute ILDs	(IIP + (AE OR ARF)) OR ((IP OR HP) + AE) OR (OP + (AE OR emergency admission OR pulse/high/moderate dose steroid*) OR AIP OR acute drug-induced ILDs OR drug-induced IP OR EP OR DM-ILD OR (IPF + (AE OR emergency admission) + pulse/high/moderate dose steroid*) OR (IIP + emergency admission + pulse/high/moderate dose steroid*) OR (IP + (emergency admission OR ARF) + pulse/high/moderate dose steroid*) OR (pulmonary fibrosis + AE + pulse/high/moderate dose steroid*) OR (pulmonary fibrosis + (emergency admission OR ARF) + pulse/high dose steroid*)	487	431	0.89 (0.85-0.91)
Broad algorithm without corticosteroid information				
AE-IP	((IPF OR IIP OR IP OR HP) + AE)	267	215	0.81 (0.75-0.85)
Acute ILDs	AIP OR acute drug-induced ILDs OR drug-induced IP OR OP OR EP OR (IIP + ARF)	198	153	0.77 (0.71-0.83)
AE-IP or Acute ILDs	((IPF OR IIP OR pulmonary fibrosis OR IP OR HP) + AE)) OR ((IIP OR IP OR HP) + ARF) OR AIP OR acute drug-induced ILDs OR drug-induced IP OR OP OR EP OR DM-ILD	519	441	0.85 (0.82-0.88)
Broad algorithm with corticosteroid information				
AE-IP	((IPF OR IIP OR IP OR HP) + AE) OR (IPF + emergency admission + pulse/high/moderate dose steroid*)	265	216	0.82 (0.76-0.86)
Acute ILDs	AIP OR acute drug-induced ILDs OR drug-induced IP OR OP OR EP OR (IIP + ARF) OR (IIP + emergency admission + pulse/high/moderate dose steroid*) OR (pulmonary fibrosis + (emergency admission OR ARF) + pulse/high dose steroid*) OR (IP + emergency admission + steroid pulse*) OR (IP + ARF + pulse/high/moderate dose steroid*)	230	176	0.77 (0.71-0.82)
AE-IP or Acute ILDs	((IPF OR IIP OR pulmonary fibrosis OR IP OR HP) + AE)) OR ((IIP OR IP OR HP) + ARF) OR AIP OR acute drug-induced ILDs OR drug-induced IP OR OP OR EP OR DM-ILD OR ((IPF OR IIP OR IP) + emergency admission + pulse/high/moderate dose steroid) OR (pulmonary fibrosis + (emergency admission OR ARF) + pulse/high dose steroid*)	527	448	0.85 (0.82-0.88)

Narrow Algorithm With Corticosteroid Information

In addition to the narrow algorithm of AE-IP without corticosteroid information, several disease names that combined IPF with AE modifiers, emergency hospitalization, and information on steroid therapy had PPVs of 0.8 or higher. A narrow algorithm with corticosteroid information with a PPV of 0.84 (95% CI, 0.78-0.88) was derived by combining these disease names (Table 7).

Broad Algorithm Without Corticosteroid Information

In addition to the narrow algorithm of AE-IP without corticosteroid information, AE-IPF had a PPV higher than 0.7, and a broad algorithm without corticosteroid information with a PPV of 0.81 (95% CI, 0.75-0.85) was derived (Table 7). 

Broad Algorithm With Corticosteroid Information

In addition to the narrow algorithm of AE-IP with corticosteroid information, AE-IPF had a PPV higher than 0.7, and a broad algorithm with corticosteroid information and a PPV of 0.82 (95% CI, 0.76-0.86) was derived (Table 7).

Algorithm for acute ILDs

Narrow Algorithm Without Corticosteroid Information

Several disease names with PPVs higher than 0.8 for acute ILDs were identified, including EP, AE-OP, acute drug-induced ILDs, and drug-induced IP + emergency admission. Combining these disease names led to the development of a narrow algorithm without corticosteroid information with a PPV of 0.82 (95% CI, 0.74-0.88; Table 7). 

Narrow Algorithm With Corticosteroid Information

In addition to the narrow algorithm of acute ILDs without corticosteroid information, several disease names that combined idiopathic IP, IP, OP, and pulmonary fibrosis with ARF, emergency hospitalization, and information on steroid therapy had PPVs higher than 0.8, and a narrow algorithm with corticosteroid information and a PPV of 0.82 (95% CI, 0.75-0.87) was derived by combining these disease names (Table 7).

Broad Algorithm Without Corticosteroid Information

Among the disease names of the narrow algorithm for acute ILDs without corticosteroid information, drug-induced IP and OP had PPVs higher than 0.7, without combinations with AE modifiers or emergency admission flags. Furthermore, acute IP and idiopathic IP + ARF had PPVs > 0.7. Combining these disease names led to the development of a broad algorithmwithout corticosteroid information with a PPV of 0.77 (95% CI, 0.71-0.83; Table 7). 

Broad Algorithm With Corticosteroid Information

In addition to the broad algorithm of acute ILDs without corticosteroid information, several disease names including idiopathic IP, pulmonary fibrosis, IP + emergency admission + information on steroid therapy, and IP + ARF + corticosteroid information had PPVs of 0.7 or higher. Combining these disease names, we finally derived a broad algorithm with corticosteroid information and a PPV of 0.77 (95% CI, 0.71-0.82; Table 7).

Algorithm for AE-IP or acute ILDs

Narrow Algorithm Without Corticosteroid Information

Several disease names with PPVs higher than 0.8 for either AE-IP or acute ILDs were identified, including AIP, acute drug-induced ILDs and IP, dermatomyositis-ILD, idiopathic IP + AE/ARF, OP + AE/emergency admission, and IP/HP + AE. We derived a narrow algorithm without corticosteroid information with a PPV of 0.89 (95% CI, 0.85-0.92) by combining these disease names (Table 7). 

Narrow Algorithm With Corticosteroid Information

In addition to the narrow algorithm of AE-IP or acute ILDs without corticosteroid information, several disease names that combined IPF, idiopathic IP, IP, and pulmonary fibrosis with AE modifiers, ARF, emergency hospitalization, and information on steroid therapy had PPVs of 0.8 or higher. A narrow algorithm with corticosteroid information and a PPV of 0.89 (95% CI, 0.85-0.91) was derived by combining these disease names (Table 7).

Broad Algorithm Without Corticosteroid Information

Among the narrow algorithms of AE-IP or acute ILDs without corticosteroid information, OP had a PPV > 0.7, without combinations with AE modifiers or emergency admission flags. In addition, IPF/pulmonary fibrosis + AE and IP/HP + ARF had PPVs > 0.7. Finally, we derived a broad algorithm without corticosteroid information and with a PPV of 0.85 (95% CI, 0.82-0.88) by combining these disease names (Table 7).

Broad Algorithm With Corticosteroid Information

In addition to the broad algorithm of AE-IP or acute ILDs without corticosteroid information, IPF/idiopathic IP/IP + emergency admission + corticosteroid information and pulmonary fibrosis + emergency admission/ARF + information on steroid therapy had PPVs of 0.7 or higher. A broad algorithm with corticosteroid information and a PPV of 0.85 (95% CI, 0.82-0.88) was derived by combining these disease names (Table 7).

## Discussion

In this study, we developed algorithms to identify patients with AE-IP and acute ILDs using Japanese administrative data, which, to the best of our knowledge, is a first. An algorithm for PAH detection could not be developed because of the small number of TPs. The PPVs for the final algorithms of AE-IP, acute ILDs, and AE-IP/acute ILDs without corticosteroid information are as follows: narrow algorithm, 0.82 (95% CI, 0.76-0.86), 0.82 (0.74-0.88), and 0.89 (0.85-0.92); broad algorithm, 0.81 (0.75-0.85), 0.77 (0.71-0.83), and 0.85 (0.82-0.88), respectively. In addition, those with corticosteroid information are as follows: narrow algorithm, 0.84 (95% CI, 0.78-0.88), 0.82 (0.75-0.87), and 0.89 (0.85-0.91); broad algorithm, 0.82 (0.76-0.86), 0.77 (0.71-0.82), and 0.85 (0.82-0.88), respectively. Despite drugs being administered and procedures being performed, the proportion of claims for them recorded in the insurance claims data was low, whereas all of them were archived in the DPC. 

The narrow and broad algorithms for AE-IP and acute ILDs derived in this study had favorable PPVs. We recently reported a validation study of AE-IPF in which the PPVs for the narrow and broad algorithms were 0.72 (95% CI, 0.62-0.81) and 0.61 (0.53-0.68), respectively [8]. This multicenter study was conducted in eight Japanese tertiary centers and focused only on AE-IPF, excluding AE of other ILDs. Furthermore, a validation study of AE-IPF performed in the United States showed a PPV of 0.621 (95% CI 0.533-0.704) [22]. Both algorithms of AE-IP derived in the present study had a higher PPV than the algorithms used in the previous studies. This may be because we did not distinguish only AE-IPF but also considered the AE of other IPs. It is often difficult to distinguish IPF from other fibrotic ILDs. However, after developing AE, patients with IPF and those with other fibrotic ILDs receive similar treatment, mainly high-dose corticosteroid therapy, and their prognosis is equally poor [25]. Therefore, we see no problem in lumping the AEs of IPF and other fibrotic ILDs together, and the algorithm developed in this study can be applied to studies of patients with AE-IP. A recent validation study examined the validity of DPC data for various respiratory diseases and found high specificity and PPVs for both IPF and non-IPF IP [9]. Both IPF (J841 for IPF in Japanese) and non-IPF (C966, D219, E85, and J841 for IPs other than IPF in Japanese and J60-J65, J67, J70, J840, M05, M06, and M30-35) IP data were extracted if one of these disease names was listed in the DPC database, and their algorithm did not include modifiers such as AE. Therefore, IP with an acute course was not considered extractable using their algorithm. In addition, the ICD-10 codes for non-IPF IPs considered in that study were more extensive than those in our study. However, the ICD-10 codes that were not included in our study denoted rare IPs and IPs with non-acute courses. Therefore, not using these ICD-10 codes for identifying AE-IP and acute ILDs was not considered a major problem. We investigated the validity of diagnosis for IP in a larger number of patients, especially those with AE-IP and acute ILDs, and developed algorithms with a high PPV.

The receipt data of hospitalized patients may be an unreliable source of information regarding the use of drugs and procedures. This is because a low percentage of these treatments and procedures are typically recorded on receipts, even if they were actually administered, whereas they are more thoroughly documented in the DPC database. The reason for this is that, under the Japanese DPC system, a fixed amount is paid based on the combination of disease and procedure instead of fee-for-service; thus, detailed claims are meaningless. It is inappropriate to use inpatient drug and procedure claims when conducting studies using receipt data.

Additional information on steroid therapy may be useful in identifying AE-IP or acute ILD cases; however, the impact of this information may depend on the algorithm used. In the present study, adding information on steroid therapy increased the PPV for most disease names. For a narrow algorithm, adding this information can increase the number of TPs without reducing the PPV and can help in the accurate identification of AE-IP or acute ILDs. In other words, algorithms based on steroid information help to extract target patients with higher sensitivity without compromising specificity. Meanwhile, for the broad algorithm, adding information on steroid therapy did not markedly increase the number of TPs. This suggests that including this information may be more helpful in identifying cases of AE-IP or acute ILDs in a limited patient population specified by researchers than in the general patient population. 

Clinical and research implications

The algorithms developed in our study could contribute to future database studies using Japanese administrative data on AE-IP and acute ILDs. Some previous studies have used thresholds of 80-85% to denote the PPVs of their algorithms as "high" [26,27], and our algorithms have PPVs close to these thresholds and are sufficient to identify patients with AE-IP and acute ILDs, rare diseases with no established treatment. Although the algorithms developed in this study generally have PPVs above 80%, many of the false-positive patients were characterized by having IP but not AE or acute ILDs, which requires caution when interpreting eligible patients. Studies that examine drug efficacy and identify drug toxicities require large sample sizes. We believe our algorithms will be useful for database studies on drug efficacy and toxicity that require large sample sizes. 

However, in this study, we did not calculate the sensitivity or specificity, but only calculated the PPV. The algorithms are not designed to include all of the target population, as there may be other patients with AE-IP and acute ILDs than those included in the present study. In other words, if the results of the present study are used in a database study, the entire population of patients with AE-IP and acute ILDs cannot be included. Therefore, further studies using random or “all possible cases” sampling are needed to calculate the sensitivity and specificity. 

Furthermore, large-scale studies are required to develop an algorithm for PAH detection, which we could not achieve due to the small number of TPs.

Strength and limitations

Our study has several strengths. First, this is the first study to develop algorithms for AE-IP and acute ILDs using Japanese administrative data. Second, we have studied a large number of patients and developed algorithms with a high PPV.

The present study has several limitations. First, it was conducted at a single tertiary hospital in Japan, which may limit the generalizability of the insurance claims data and DPC in Japan as a whole. Furthermore, the results of this study are naturally not applicable to patients in other countries. Therefore, our results should be validated in other Japanese medical institutions and other countries. Second, AE-IP, acute ILDs, and PAH are sometimes difficult to accurately diagnose, and the decision of the two respiratory physicians depended on the tests conducted by the physicians who treated patients. Therefore, misclassification cannot be completely ruled out. Third, although patients with acute ILDs comprise a heterogeneous population, we considered them as a single group because it was clinically challenging to precisely identify the underlying cause in many cases. Therefore, the findings for this group should be interpreted with caution.

## Conclusions

The algorithms developed in this study had a high PPV for identifying patients with AE-IP and acute ILDs. Incorporating information on steroid treatment may enhance the specificity of patient selection for AE-IP and acute ILD cases. However, the recording of administered drugs, procedures, and tests in hospital claim data was found to be infrequently documented. Therefore, reliance on claim data alone for these data is not recommended for inpatient studies. These insights are valuable for future database research in Japan. The algorithms we have created can be used in future epidemiological studies using Japanese administrative data to investigate the efficacy and toxicity of treatments in patients with AE-IP and acute ILDs.

## Appendices

Appendix A describes the checklist of reporting criteria for studies validating health administrative data algorithms (Table 8).

**Table 8:** Checklist of reporting criteria for studies validating health administrative data algorithms

	YES	NO	UNCERTAIN	NOT APPLICABLE
TITLE, KEYWORDS, ABSTRACT				
Identify article as study of assessing diagnostic accuracy	✔︎			
Identify article as study of administrative data	✔︎			
INTRODUCTION				
State disease identification & validation one of goals of study	✔︎			
METHODS				
Participants in validation cohort				
Describe validation cohort (Cohort of patients to which reference standard was applied)	✔︎			
∙ Age	✔︎			
∙ Disease	✔︎			
∙ Severity				✔︎
∙ Location/Jurisdiction	✔︎			
Describe recruitment procedure of validation cohort				
∙ Inclusion criteria	✔︎			
∙ Exclusion criteria				✔︎
Describe patient sampling (random, consecutive, all, etc.)	✔︎			
Describe data collection	✔︎			
∙ Who identified patients and did the selection adhere to patient recruitment criteria?	✔︎			
∙ Who collected data	✔︎			
∙ A priori data collection form	✔︎			
∙ Disease classification	✔︎			
∙ Split sample (i.e. re-validation using a separate cohort)				✔︎
Test Methods:				
Describe number, training and expertise of persons reading reference standard	✔︎			
If >1 person reading reference standard, quote measure of consistency (e.g. kappa)	✔︎			
Blinding of interpreters of reference standard to results of classification by administrative data e.g. Chart abstractor blinded to how that chart was coded	✔︎			
Statistical Methods:				
Describe methods of calculating/comparing diagnostic accuracy	✔︎			
RESULTS:				
Participants:				
Report when study done, start/end dates of enrollment	✔︎			
Describe number of people who satisfied the inclusion/exclusion criteria	✔︎			
Study flow diagram				✔︎
Test results:				
Report distribution of disease severity				✔︎
Report cross-tabulation of index tests by results of reference standard	✔︎			
Estimates:				
Report at least 4 estimates of diagnostic accuracy				✔︎
Diagnostic Accuracy Measures Reported:				
∙ Sensitivity				✔︎
∙ Spec				✔︎
∙ PPV	✔︎			
∙ NPV				✔︎
∙ Likelihood ratios				✔︎
∙ Kappa		✔︎		
∙ Area under the ROC				✔︎
curve / c-statistic				
∙ Accuracy/agreement	✔︎			
∙ Other (specify)				✔︎
Report accuracy for subgroups (e.g. age, geography, different sex, etc.)				✔︎
If PPV/NPV reported, ratio of cases/controls of validation cohort approximate prevalence of condition in the population			✔︎	
Report 95% confidence intervals for each diagnostic measure	✔︎			
DISCUSSION:				
Discuss the applicability of the validation findings	✔︎			
